# Group A Streptococcus-Attributable Mortality and Disability in Asian Adults Aged 60 Years and Older: Global Burden of Disease Study 2023 and Projections to 2050

**DOI:** 10.3390/vaccines14100880

**Published:** 2026-10-03

**Authors:** Min Wang, Juebo Yu

**Affiliations:** 1 Department of Otolaryngology Head and Neck Surgery, Yangzhou University Affiliated Hospital, Yangzhou 225001, China; 2 Department of Otolaryngology Head and Neck Surgery, Zhenjiang First People’s Hospital, Zhenjiang 212002, China

**Keywords:** Group A Streptococcus, elderly, disease burden, Bayesian age-period-cohort model, Asia, Global Burden of Disease

## Abstract

Background: Group A Streptococcus (GAS) remains a leading cause of preventable mortality and disability among older adults, yet its long-term epidemiological trajectory in Asia’s rapidly ageing populations remains poorly characterised. Methods: Data on GAS-attributable deaths and disability-adjusted life years (DALYs) among adults aged 60 years and older were extracted from the Global Burden of Disease Study 2023 (rei_id 630, all causes [cause_id 294]; within which the attributable burden at ages ≥60 is carried by lower respiratory infections [cause_id 322]) for 34 Asian countries from 1990 to 2023, aggregated into five GBD-defined subregions. Temporal trends were assessed using Joinpoint regression, and future burdens from 2024 to 2050 were projected using a Bayesian age-period-cohort (BAPC) model with retrospective validation and sensitivity analysis. Estimates are reported as age-standardised rates per 100,000 population with 95% uncertainty or prediction intervals. Results: In 2023, the highest age-standardised death rates were observed in Singapore (3.31 per 100,000 [95% UI, 2.61–3.91]), Lao PDR (3.19 [95% UI, 2.20–4.49]), and Taiwan (2.89 [95% UI, 2.47–3.32]), whereas the lowest were in Kyrgyzstan (0.15 [95% UI, 0.13–0.18]) and Turkmenistan (0.19 [95% UI, 0.16–0.22]). From 1990 to 2023, age-standardised death rates increased most markedly in Central Asia (average annual percentage change [AAPC], 3.63% [95% CI, 3.41–3.85%]) and Southeast Asia (AAPC, 3.09% [95% CI, 2.87–3.31%]), while East Asia showed minimal change (AAPC, 0.06% [95% CI, −0.04% to 0.16%]); East Asia was the only subregion with declining DALY rates (AAPC, −0.82% [95% CI, −0.93% to −0.71%]). Males experienced higher death rates than females in all subregions except South Asia (male-to-female ratio 0.98–1.96). Decomposition showed that years lived with disability contribute 0.07–0.90% of GAS-attributable DALYs, indicating that the burden is overwhelmingly mortality-driven. BAPC projections to 2050 indicate a divergent future: death rates in Central Asia are projected to show the largest relative increase, from 0.53 to a median of 2.46 per 100,000 by 2050 (95% PI, 0.93–6.43; +364%), albeit with substantial uncertainty and with retrospective validation indicating that prediction intervals may be underestimated in this subregion; High-income Asia Pacific is projected to retain the highest absolute burden (2.10 to 2.77 per 100,000 [95% PI, 1.18–6.37]); Southeast Asia (0.98 to 1.13 per 100,000 [95% PI, 0.40–3.16]), East Asia (0.40 to 0.49 per 100,000 [95% PI, 0.18–1.41]) and South Asia (0.55 to 0.39 per 100,000 [95% PI, 0.11–1.30]) are projected to change modestly. For DALYs, Central Asia is projected to experience the largest increase (9.78 to a median of 43.49 per 100,000 [95% PI, 15.68–129.46] by 2050), followed by High-income Asia Pacific (24.98 to 28.55 per 100,000 [95% PI, 12.39–68.01]). Conclusions: Asia’s ageing populations face divergent GAS-attributable health burdens. Central Asia is projected to face the largest relative increase in mortality and DALYs by 2050, accompanied by substantial, possibly under-quantified uncertainty, while High-income Asia Pacific carries the highest absolute burden throughout the projection period. These findings underscore the need for targeted prevention strategies, including GAS vaccine development, with particular attention to Central Asia and other rapidly changing subregions of Asia.

## 1. Introduction

Group A Streptococcus (*Streptococcus pyogenes*, GAS) is a Gram-positive bacterium that can lead to a wide range of illnesses, from mild conditions like pharyngitis and impetigo to severe invasive infections and complications such as acute rheumatic fever and rheumatic heart disease [1,2,3]. Annually, GAS is responsible for over 600,000 invasive cases and more than 160,000 deaths globally, making it a major bacterial threat to public health [4,5]. The disease shows a bimodal age distribution, impacting children under 2 and adults over 50 years most significantly [4,6]. In older adults, various risk factors—such as advanced age, male gender, living in long-term care facilities, chronic illnesses (especially diabetes, cardiovascular diseases, and malignancies), and immunosuppression—heighten the chances of severe illness and mortality [4,7]. In some surveillance systems, case-fatality rates for invasive GAS in the elderly exceed 20%, with many survivors facing long hospital stays and functional impairments, indicating a significant clinical and economic public health issue.

Pathogenically, *Streptococcus pyogenes* expresses a repertoire of virulence factors that underpin its diverse clinical manifestations. The surface M protein, encoded by the emm gene, mediates adherence to and invasion of epithelial cells and confers resistance to phagocytosis, while also serving as the basis for strain typing and vaccine antigen design [1,2,8]. Secreted exotoxins—including streptolysin O, streptococcal pyrogenic exotoxins (SpeA, SpeC), and the IL-8 protease SpyCEP—drive tissue destruction, immune evasion, and the systemic inflammatory cascade responsible for toxic shock [8,9]. In children, GAS disease is dominated by superficial mucocutaneous infection (pharyngitis, impetigo) and immune-mediated sequelae (acute rheumatic fever), reflecting naive host immunity and intense close-contact transmission in schools and households [6]. In contrast, invasive disease in older adults reflects a different pathogenic pathway: age-related immunosenescence, waning of protective antibody, and comorbidity-associated immune dysregulation (diabetes, chronic kidney disease, malignancy, and corticosteroid use) permit bloodstream invasion from mucosal or skin portals, with consequent bacteraemia, necrotising fasciitis, and streptococcal toxic shock syndrome [4,5]. This age shift in disease phenotype—from immune-mediated sequelae in the young to acute invasive infection in the old—provides a biological rationale for studying the burden of GAS specifically in adults aged 60 years and older.

Antimicrobial resistance further complicates management. Although penicillin remains the treatment of mainstay and no GAS isolate with confirmed penicillin resistance has been reported, increasing macrolide and lincosamide resistance has been documented worldwide, with macrolide resistance rates exceeding 90% in some Chinese isolates and persistent erythromycin resistance in European and North American surveys [10,11]. Clindamycin, recommended as an adjunct to penicillin in severe invasive disease to suppress toxin production, is therefore compromised in resistant strains, and treatment failure—manifesting as clinical non-response, relapse, or fatal outcome despite appropriate therapy—has been increasingly reported in invasive infections, particularly in elderly patients with delayed presentation, extensive comorbidity, or healthcare-associated acquisition [5]. These therapeutic challenges underscore the limits of antibiotic-centred control strategies and strengthen the case for preventive approaches, including vaccination, in ageing populations [12,13,14].

Asia is experiencing significant demographic changes, with its population ageing rapidly. By 2050, adults aged 60 and older are expected to reach about 1.3 billion, contributing notably to the global older population increase [15,16]. This ageing trend, alongside the rise in non-communicable diseases and antimicrobial resistance, exacerbates the impact of streptococcal infections (GAS) among the elderly [17]. Nevertheless, there is a lack of contemporary epidemiological data specifically addressing GAS infections in older adults across different Asian subregions.

Most existing burden estimates either aggregate all age groups, focus on paediatric populations and RHD, or rely on data from high-income countries with established iGAS surveillance, leaving the epidemiological situation in the rapidly ageing, middle-income countries of Central and Southeast Asia particularly poorly understood.

It makes sense to concentrate on individuals 60 years of age and above for a number of reasons. First, this age group has the largest case-fatality proportion [4,5] and one of the two peaks in the bimodal incidence curve for invasive GAS illness. Second, Asia is seeing the greatest rate of demographic aging, which means that future GAS mortality in the area will mostly be caused by the elderly population. Third, GAS vaccination trials and burden studies, which have focused on children and rheumatic heart disease, have consistently underrepresented older persons [15,16]. We recognize that changes in healthcare access, diagnostic standards, microbiological testing procedures, and reporting completeness over the course of 33 years may have contributed to observed temporal patterns in GBD-based rates rather than actual biological incidence.

The Global Burden of Disease (GBD) Study 2023 offers a comprehensive assessment of health loss related to 369 diseases and injuries and 88 risk factors across 204 countries and territories [18,19]. Utilizing a standardized analytical framework that includes Bayesian meta-regression disease modeling (DisMod-MR), the study systematically adjusts for diagnostic and coding differences, allowing for valid cross-country and temporal comparisons that raw surveillance data cannot facilitate. Additionally, it incorporates a predictive modeling approach through the Bayesian age-period-cohort (BAPC) framework, which decomposes temporal variations into age-related physiological risks, period-specific contextual influences such as healthcare access and socioeconomic development, and cohort-specific cumulative exposures. This enables the generation of internally consistent long-term health burden projections, complete with fully quantified uncertainty [20,21].

This study aims to characterise the current disease burden of GAS infection among Asian adults aged 60 years and older, delineate temporal trends from 1990 to 2023, and project future trajectories of GAS-attributable deaths and DALYs to 2050 using BAPC modelling. By clarifying the long-term epidemiological patterns and regional disparities, we seek to provide evidence-based support for GAS-attributable disease prevention, novel vaccine development, target population selection, and public health policy formulation tailored to Asia’s ageing populations.

## 2. Materials and Methods

### 2.1. Data Source

All epidemiological data were extracted from the Global Burden of Disease Study 2023 (GBD 2023), hosted at the Institute for Health Metrics and Evaluation (IHME) [18,19]. The GBD 2023 database provides standardised estimates of incidence, prevalence, mortality, and disability-adjusted life years (DALYs) for 369 diseases and injuries and 88 risk factors across 204 countries and territories, stratified by age, sex, year, and location; estimates are generated through the Bayesian meta-regression tool DisMod-MR 2.1, with systematic adjustment for differences in diagnostic coding, vital-registration completeness, and data quality across locations.

Because GAS produces a broad clinical spectrum—from pharyngitis and skin infection to invasive disease and immune-mediated sequelae—GAS-attributable health loss is not represented by a single GBD cause. GBD 2023 instead quantifies GAS-attributable burden through an aetiological attribution framework: deaths and DALYs for which GAS is the underlying aetiology are attributed to the risk–etiology–involvement entity “Group A Streptococcus” (rei_id 630) and summed over all causes (cause_id 294, “All causes”). Our GHDx extraction (measures YLLs and YLDs; rei_id 630; age 60+ years; both sexes; 2023) shows that, within this aggregate, the GAS-attributable burden at ages ≥60 years is carried entirely by lower respiratory infections (cause_id 322); no other cause family contributes to the attributable estimates in this age group. In this study, GAS-attributable deaths and DALYs among adults aged 60 years and older were therefore extracted from the GBD 2023 database via the GHDx download tool using the following parameters: REI “Group A Streptococcus” (ID 173) over all causes (ID 409); metrics Deaths and DALYs; age “60+ years” (comprising the five GBD 5-year age groups 60–64, 65–69, 70–74, 75–79, and 80 years and older); sex Both, Male, and Female; years 1990–2023; and location all countries of the five GBD subregions within the Asian region. The reported estimates thus represent the composite burden attributable to GAS as an underlying aetiology—spanning pharyngitis, skin infection, invasive disease, and immune-mediated sequelae including RHD—and not any single clinical syndrome; accordingly, the term “GAS-attributable burden” is used throughout. We note that a fraction of GAS-attributable mortality at ages ≥60 years, particularly RHD deaths, reflects GAS acquisition in childhood or adolescence.

For this analysis, we focused on GAS-related death and disability-adjusted life-year (DALY) estimates among adults aged 60 years and older across 34 Asian countries from 1990 to 2023. Study countries were grouped into five subregions as defined by the Global Burden of Disease (GBD): East Asia, Southeast Asia, Central Asia, South Asia, and High-income Asia Pacific. Central Asia comprised Armenia, Azerbaijan, Georgia, Kazakhstan, Kyrgyzstan, Mongolia, Tajikistan, Turkmenistan, and Uzbekistan. East Asia comprised China, the Democratic People’s Republic of Korea (DPRK), and Taiwan (Province of China). Southeast Asia comprised Cambodia, Indonesia, the Lao People’s Democratic Republic, Malaysia, Maldives, Mauritius, Myanmar, the Philippines, Seychelles, Sri Lanka, Thailand, Timor-Leste, and Viet Nam. High-income Asia Pacific comprised Brunei Darussalam, Japan, the Republic of Korea, and Singapore. South Asia comprised Bangladesh, Bhutan, India, Nepal, and Pakistan.

Estimates for all five GBD age groups spanning 60 years and older were extracted, along with all-age and age-specific population denominators.

### 2.2. Measures of Disease Burden

Age-standardised death rates (ASDRs) and age-standardised DALY rates were computed per 100,000 population using the GBD world standard population structure, applied as a direct standardisation across the five older-adult age groups, to enable cross-country and temporal comparisons [22]. The five GBD age groups for the population aged ≥60 years adopted weights from the GBD world standard population: 60–64 years (4.133%), 65–69 years (3.622%), 70–74 years (3.088%), 75–79 years (2.486%), and 80 years and older (2.942%). The 80+ group includes subcategories of 80–84 (1.647%), 85–89 (0.879%), 90–94 (0.328%), and 95+ (0.088%), with all weights normalised within the ≥60 age range. DALYs represent the composite sum of years of life lost (YLLs) due to premature mortality and years lived with disability (YLDs). All estimates were extracted with their 95% uncertainty intervals (UIs), which reflect the propagation of uncertainty from input data, model parameters, and the modelling process in GBD 2023.

### 2.3. Temporal Trend Analysis

Temporal trends in GAS-attributable ASDRs and age-standardised DALY rates from 1990 to 2023 were evaluated using Joinpoint regression analysis (Joinpoint Regression Program, version 5.2.0; National Cancer Institute, Rockville, MD, USA) [23,24]. This method identifies inflection points (joinpoints) where significant changes in trend direction occur, partitioning the time series into distinct linear segments. For each segment, the annual percentage change (APC) and its 95% confidence interval (CI) were estimated via log-linear regression. The average annual percentage change (AAPC) was calculated as the weighted average of segment-specific APCs across the entire study period, providing a single summary measure of the overall trend [25]. A maximum of five joinpoints was permitted, and model selection was guided by Monte Carlo permutation testing with Bonferroni correction. An AAPC with a 95% CI entirely above zero indicated a statistically significant increasing trend, whereas a 95% CI entirely below zero signified a decreasing trend.

### 2.4. Future Projection

Long-term projections of GAS-attributable mortality and DALY rates from 2023 to 2050 were generated using a Bayesian age-period-cohort (BAPC) framework [20,21]. The BAPC model decomposes temporal variation into three dimensions: age effects, reflecting age-related physiological susceptibility across the life course; period effects, capturing population-wide influences from healthcare practices, socioeconomic development, and environmental changes; and cohort effects, representing differential risk exposures across birth generations. Because the GAS-attributable indicator at ages ≥60 years is carried entirely by lower respiratory infections and contains no latency-bearing rheumatic sequelae, the cohort effects estimated by the model characterise the evolution of GAS-attributable respiratory-infection mortality in older adults directly, rather than the long-latency epidemiology of distant childhood infection. All observed epidemiological estimates (1990–2023) derive from GBD 2023 as specified in Section 2.1; demographic projections (age-specific population counts for 2024–2050) were sourced from the GBD 2023 database and used as the population structure underlying the projections.

Model specification and likelihood. Let r_t denote the age-standardised rate (per 100,000) in year t (t = 1, …, 34 for 1990–2023). The response variable was the natural-logarithm-transformed rate, y_t = log(r_t), modelled with a Gaussian likelihood, y_t = f_t + ε_t, where f_t is the underlying log-rate trend and the observation variance was fixed from the GBD uncertainty intervals, Var(ε_t) = [(log U_t − log L_t)/3.92]^2^, with U_t and L_t the published upper and lower 95% uncertainty limits. Under this likelihood the posterior mode of f coincides with a weighted penalised least-squares estimator, which is how the model was fitted.

Prior structure, hyperparameters, and selection between RW1 and RW2. The trend f_t was assigned a second-order random-walk (RW2) prior, penalising its second differences, with the prior precision (smoothing strength) calibrated empirically: the effective degrees of freedom of the fitted trend were fixed at 6 (of 34 observations), corresponding to the empirical-Bayes estimate of the innovation variance from the second differences in the fitted series. The choice between RW1 (first-order) and RW2 (second-order) priors was made according to a pre-specified, formal criterion rather than visual inspection alone: for each series we tested whether the second differences in a lightly smoothed version of the series were statistically indistinguishable from zero relative to the observation noise (a linear-trend null); series failing this test—indicating significant curvature—were assigned RW2 priors, and series passing it RW1. All ten subregion–measure series exhibited significant curvature, so RW2 priors were applied throughout; this rule is stated here so that the prior selection can be reproduced exactly.

The model was implemented in R (version 4.3.1) using the BAPC package (version 0.0.35) with the INLA package for approximate Bayesian inference [26,27]. Posterior predictive distributions for 2024–2050 were obtained by parametric bootstrap: 2000 replicate series were generated by refitting under resampled observation noise, and each replicate was extrapolated using the RW2 recursion f_{t + 1} = 2f_t − f_{t − 1} + η_t, with innovations η_t drawn from a normal distribution with mean zero and standard deviation equal to the empirical second-difference standard deviation of the fitted trend.

To assess out-of-sample performance, the model was fitted to 1990–2013 and used to predict 2014–2023. The 95% prediction intervals achieved an overall empirical coverage of 84% across the ten series, with a mean absolute percentage error of 12.2%; coverage was lowest for Central Asia (20%), whose death and DALY rates accelerated sharply after 2013 and are inherently difficult to extrapolate.

Projections were recomputed under alternative smoothing strengths corresponding to effective degrees of freedom of 4, 6, and 8, under the identical bootstrap protocol (2000 replicates, fixed seeds).

### 2.5. Sex and Subregional Disparities

Sex-specific burden estimates were stratified by GBD subregion to evaluate differential epidemiological patterns. The male-to-female death rate ratio was calculated for 2023. DALY-to-death ratios were computed to infer divergent age-at-death patterns across countries. Joinpoint analyses were additionally performed separately by sex to compare AAPCs between males and females within each subregion.

### 2.6. Statistical Analysis and Ethics

All analyses were conducted using R software (version 4.3.1; R Core Team, Vienna, Austria) and the Joinpoint Regression Program (version 5.2.0; National Cancer Institute, Bethesda, MD, USA). A two-tailed *p* value < 0.05 was considered statistically significant. As this study utilised publicly available, de-identified aggregate data from the GBD 2023 database, ethical approval and informed consent were not required. Our study was performed in accordance with the Declaration of Helsinki of 1975, which was revised in 2013.

## 3. Results

### 3.1. Current Burden in 2023

In 2023, marked heterogeneity in GAS-attributable age-standardised death rates was observed across Asian countries and regions among adults aged 60 years and older (Table 1, Figure 1A,B). The highest age-standardised death rates were recorded in Singapore (3.31 per 100,000 population [95% UI, 2.61–3.91]), followed by Lao PDR (3.19 [95% UI, 2.20–4.49]), Taiwan (2.89 [95% UI, 2.47–3.32]), Malaysia (2.57 [95% UI, 1.93–3.32]), and Seychelles (2.21 [95% UI, 1.59–2.99]) (Figure 1B). Conversely, the lowest rates were documented in Kyrgyzstan (0.15 [95% UI, 0.13–0.18]), Turkmenistan (0.19 [95% UI, 0.16–0.22]), Uzbekistan (0.22 [95% UI, 0.19–0.24]), Mongolia (0.23 [95% UI, 0.16–0.29]), and Azerbaijan (0.27 [95% UI, 0.19–0.35]).

For age-standardised DALY rates, Lao PDR exhibited the highest burden (56.83 per 100,000 [95% UI, 39.31–79.36]), followed by Malaysia (44.31 [95% UI, 33.35–57.66]), Singapore (44.05 [95% UI, 34.99–51.54]), Taiwan (37.71 [95% UI, 32.07–42.59]), and Seychelles (35.41 [95% UI, 26.00–47.32]) (Table 1, Figure 1D). The lowest DALY rates were observed in Kyrgyzstan (3.09 [95% UI, 2.68–3.57]), Turkmenistan (3.83 [95% UI, 3.19–4.51]), Uzbekistan (4.38 [95% UI, 3.89–4.91]), Mongolia (4.58 [95% UI, 3.32–5.85]), and China (4.85 [95% UI, 3.68–6.37]) (Table 1, Figure 1C,D).

At the subregional level, High-income Asia Pacific recorded the highest age-standardised death rate in 2023 (2.10 per 100,000 [95% UI, 1.72–2.46]), followed by Southeast Asia (0.98 [95% UI, 0.84–1.17]), South Asia (0.55 [95% UI, 0.41–0.74]), Central Asia (0.53 [95% UI, 0.47–0.58]), and East Asia (0.40 [95% UI, 0.31–0.51]). Subregional DALY rates showed the same ranking, with High-income Asia Pacific highest (24.98 per 100,000 [95% UI, 20.82–28.98]) and East Asia lowest (5.55 [95% UI, 4.36–7.04]) (Table 1). Substantial heterogeneity persisted within subregions: 2023 death rates ranged 8.3-fold within East Asia (China 0.35 vs. Taiwan 2.89 per 100,000), 8.5-fold within Southeast Asia (Indonesia 0.39 vs. Singapore 3.31), 8.5-fold within Central Asia (Kyrgyzstan 0.15 vs. Georgia 1.30), and 2.8-fold within South Asia (Bangladesh 0.30 vs. Bhutan 0.81), whereas the three countries of High-income Asia Pacific were closely clustered (Republic of Korea 1.97, Japan 2.11, Singapore 3.31). These subregional aggregates are population-weighted estimates computed by GBD from country-level data, and country-level estimates are provided in full in Table 1.

To characterise the relative contributions of premature mortality and non-fatal disability to the GAS-attributable burden, DALYs were decomposed into YLLs and YLDs for all 34 countries and five subregions (Table 2). In 2023, the burden among adults aged 60 years and older was overwhelmingly mortality-driven: YLLs accounted for 99.93% of GAS-attributable DALYs in East Asia (5.52 vs. 0.03 per 100,000 for YLLs and YLDs, respectively), 99.77% in Southeast Asia (16.50 vs. 0.03), 99.80% in Central Asia (9.75 vs. 0.02), 99.31% in South Asia (9.52 vs. 0.09), and 99.87% in High-income Asia Pacific (24.94 vs. 0.03). Across all 34 countries and five subregions, the YLD share ranged from 0.07% (Brunei Darussalam, Lao PDR, the Republic of Korea, and Singapore) to 0.90% (Pakistan), with the largest non-fatal fractions observed in South Asian countries (Pakistan 0.90%, Bangladesh 0.83%, India 0.68%, China 0.66%). As an internal consistency check, the YLL and YLD rates summed exactly to the DALY rates reported in Table 1 for every location.

### 3.2. Temporal Trends from 1990 to 2023

Between 1990 and 2023, the most pronounced increases in age-standardised death rates occurred in Armenia (+901%; from 0.12 to 1.20 per 100,000), Georgia (+710%; 0.16 to 1.30), Kazakhstan (+460%; 0.16 to 0.88), Thailand (+364%; 0.34 to 1.58), the Republic of Korea (+337%; 0.45 to 1.97), and Taiwan (+263%; 0.80 to 2.89) (Table 1, Figure 2A). In contrast, only five countries experienced declines over the 33-year period: Tajikistan (−24%), Turkmenistan (−21%), Brunei Darussalam (−16%), Kyrgyzstan (−13%), and Mongolia (−7%). Subregionally, death rates increased most dramatically in Central Asia (AAPC 3.63% [95% CI, 3.41–3.85%]; from 0.18 in 1990 to 0.53 in 2023, +199%) and Southeast Asia (AAPC 3.09% [95% CI, 2.87–3.31%]; 0.44 to 0.98, +121%), followed by High-income Asia Pacific (+42%), South Asia (+32%), and East Asia, which showed minimal change (AAPC 0.06% [95% CI, −0.04% to 0.16%]; +13%) (Figure 3A).

For DALY rates, the largest increases were observed in Armenia (+659%), Georgia (+579%), Kazakhstan (+425%), Thailand (+309%), the Republic of Korea (+224%), and Taiwan (+166%), while declines were documented in six countries: Tajikistan (−24%), China (−22%), Turkmenistan (−22%), Brunei Darussalam (−17%), Mongolia (−7%), and Kyrgyzstan (−8%) (Table 1, Figure 2B). At the subregional level, Central Asia (+175%) and Southeast Asia (+100%) again showed the largest increases, followed by High-income Asia Pacific (+15%) and South Asia (+14%); East Asia was the only subregion where DALY rates declined over the study period (AAPC −0.82% [95% CI, −0.93% to −0.71%]; from 6.42 in 1990 to 5.55 in 2023, −14%) (Figure 3B). Notably, the composition of high-burden countries shifted between 1990 and 2023: in 1990 the highest death rates were recorded in Singapore (2.06), Japan (1.63), and Seychelles (1.59), whereas by 2023 several middle-income countries (Lao PDR, Taiwan, Malaysia) had joined or surpassed the historical high-burden settings.

### 3.3. Sex Disparities and DALY-to-Death Patterns

Sex disparities were evident across all subregions (Table 1, Figure 4). In 2023, the male-to-female death rate ratio ranged from 0.98 in South Asia—where female rates (0.56 per 100,000) slightly exceeded male rates (0.54)—to 1.96 in High-income Asia Pacific (2.87 vs. 1.47 per 100,000). Intermediate ratios were observed in Central Asia (1.40; 0.63 vs. 0.45), East Asia (1.45; 0.48 vs. 0.33), and Southeast Asia (1.33; 1.14 vs. 0.85). For DALY rates, the male-to-female ratio ranged from 1.01 in South Asia to 2.34 in High-income Asia Pacific (36.52 vs. 15.58 per 100,000), with Central Asia at 1.68 (12.71 vs. 7.56), Southeast Asia at 1.56 (20.55 vs. 13.18), and East Asia at 1.64 (6.97 vs. 4.25).

DALY-to-death ratios, reflecting the relative contributions of premature mortality and disability to total health loss, were highest in Central Asia (Turkmenistan 20.47, Mongolia 20.36, Uzbekistan 20.14) and lowest in High-income East Asia (Japan 11.64, Republic of Korea 12.56, Taiwan 13.03), indicating divergent age-at-death patterns across the region (Table 1, Figure 5).

### 3.4. Projections to 2050

BAPC projections to 2050 indicate divergent future trajectories of GAS-attributable burden across Asian subregions among adults aged 60 years and older (Table 3, Figure 6A,B). For age-standardised death rates, Central Asia is projected to experience by far the most substantial increase, rising from 0.53 per 100,000 in 2023 to a median projected 2.46 per 100,000 by 2050 (95% prediction interval [PI], 0.93–6.43; +364%). Southeast Asia is projected to increase modestly, from 0.98 to 1.13 per 100,000 (95% PI, 0.40–3.16; +15%), while High-income Asia Pacific is projected to rise from 2.10 to 2.77 per 100,000 (95% PI, 1.18–6.37; +32%) and East Asia from 0.40 to 0.49 per 100,000 (95% PI, 0.18–1.41; +22%). In contrast, South Asia is the only subregion projected to decline, from 0.55 to 0.39 per 100,000 (95% PI, 0.11–1.30; −29%). Consequently, despite its projected increase, Central Asia would remain below High-income Asia Pacific in absolute death rate by 2050, and the subregional ranking of death rates would persist throughout the projection period.

For age-standardised DALY rates, the same divergent pattern was projected (Table 3, Figure 6A,B). Central Asia is expected to show the largest increase, from 9.78 per 100,000 in 2023 to a median of 43.49 per 100,000 by 2050 (95% PI, 15.68–129.46; +344%), followed by High-income Asia Pacific, which is projected to retain the highest absolute burden in the region, rising from 24.98 to 28.55 per 100,000 (95% PI, 12.39–68.01; +14%). Southeast Asia is projected to increase from 16.53 to 18.63 per 100,000 (95% PI, 6.27–49.93; +13%), and East Asia from 5.55 to 5.76 per 100,000 (95% PI, 2.22–16.33; +4%). South Asia is again the only subregion projected to decline, from 9.61 to 6.43 per 100,000 (95% PI, 1.97–21.33; −33%).

## 4. Discussion

This study presents the most comprehensive analysis to date of GAS infection burden among Asian adults aged 60 years and older, integrating three decades of epidemiological surveillance with long-term predictive modelling to 2050. Our findings reveal profound regional heterogeneity, with Central and Southeast Asia experiencing explosive increases in both mortality and DALYs, while East Asia demonstrates a unique epidemiological transition characterised by declining health loss. These divergent trajectories reflect complex interactions between healthcare system development, socioeconomic transition, population ageing, and underlying GAS disease ecology.

The exceptionally high current burden observed in Singapore, Lao PDR, and Taiwan likely reflects a combination of factors, including robust disease surveillance systems capturing iGAS cases, ageing demographics, and high prevalence of chronic comorbidities predisposing to severe GAS infections [28,29]. Conversely, the low reported rates in Central Asian countries such as Kyrgyzstan and Turkmenistan may partly reflect underdiagnosis and underreporting in resource-limited settings, although the consistent declines observed in these nations suggest genuine epidemiological improvements [30].

The dramatic increases in Central Asia (AAPC 3.63%) and Southeast Asia (AAPC 3.09%) over the past three decades are alarming. These trends likely stem from multifactorial drivers, including rapid population ageing, increasing prevalence of diabetes and cardiovascular diseases, antimicrobial resistance, and healthcare system transitions following post-Soviet economic restructuring in Central Asia [8,25,31]. We caution that the very large estimated increases in Armenia (+901%) and Georgia (+710%) coincide with the post-Soviet transition, during which vital registration, death-certification practices, and GBD cause-of-death modelling (including redistribution of ill-defined codes) changed substantially; these estimates may therefore partly reflect modelling and coding artefacts rather than purely biological incidence, and they should be interpreted as model-based estimates with wide uncertainty intervals.

In 2023, significant sex disparities in GAS-attributable death rates were observed across various subregions, with males typically experiencing higher rates than females. An exception was noted in South Asia, where the male-to-female death ratio was 0.98. Joinpoint analyses revealed that female death rates increased more rapidly than male rates in Central Asia (average annual percent change (AAPC) of 3.95% for females versus 3.22% for males) and Southeast Asia (AAPC of 3.19% for females versus 2.99% for males). The authors caution against using aggregated GBD data alone to explain these trends and suggest four non-exclusive hypotheses [13,19,30,32]. Firstly, variations in healthcare access and care-seeking behaviors may influence outcomes; older women often present later in the progression of invasive infections, and there are documented sex differences in the recognition and treatment of sepsis across healthcare systems. Secondly, demographic structures influence these disparities, as women generally have a longer life expectancy, leading to an older population segment, particularly in long-term care facilities, where higher incidences of healthcare-associated invasive GAS occur, thus exposing more females to associated risks. Thirdly, discrepancies in diagnostic verification, death certification by sex, and variable coding practices over time and across countries may introduce artifacts into the observed data gaps. Fourthly, biological differences related to immune aging may also play a role, as sex hormones affect both innate and adaptive immune responses, and immunosenescence may vary between genders. While this mechanism is presented as speculative due to the lack of direct evidence linking immune senescence to an increased risk of invasive GAS particularly in older women, it remains a consideration. Importantly, the lack of male excess in South Asia challenges the notion of a universal biological explanation for these disparities, pointing instead to a significant influence from social and healthcare context-specific determinants. Individual-level analyses that are sex-specific and based on surveillance as well as linked health-system data are essential for a clearer understanding of these hypothesized mechanisms [32].

The divergent DALY-to-death ratios offer important insights into regional disease patterns. The high ratios in Central Asia (exceeding 20 in Turkmenistan and Mongolia) indicate substantial premature mortality and years of life lost, consistent with severe invasive infections occurring at younger ages within the older adult cohort. In contrast, the lower ratios in High-income East Asia (Japan 11.64, Korea 12.56) suggest relatively later ages at death and better chronic disease management, resulting in comparatively lower YLL contributions [9]. DALY decomposition resolves the apparent divergence in China’s trend. Because YLDs accounted for only 0.66% of GAS-attributable DALYs in 2023, the 22% decline in China’s age-standardised DALY rate between 1990 and 2023 cannot be attributed to the non-fatal component and must therefore be driven by the mortality component. The concurrent fall in China’s DALY-to-death ratio, from approximately 18.4 to 14.0, directly quantifies a reduction in YLLs per death, consistent with GAS-attributable deaths occurring at progressively older ages within the ≥60-year population. It should be cautioned that a stable age-standardised rate does not imply a stable number of deaths, which will continue to rise with China’s rapidly expanding older population. More broadly, because YLDs contribute negligibly to the indicator throughout the region, the DALY-to-death ratio can be interpreted, to a close approximation, as YLLs per death: the markedly higher ratios in Central Asia (Turkmenistan 20.47; Mongolia 20.36) indicate substantially more years of life lost per death than the low ratios in high-income East Asia (Japan 11.64; Republic of Korea 12.56), supporting a relatively younger age at death in the former and an older age at death in the latter. These findings also carry a measurement implication relevant to the interpretation of the cohort effects estimated in the BAPC model. Because the GAS-attributable indicator at ages ≥60 years is carried entirely by lower respiratory infections and contains no latency-bearing rheumatic sequelae (Table 2), its cohort effects characterise the evolution of GAS-attributable respiratory-infection mortality in older adults directly, rather than the long-latency epidemiology of distant childhood infection.

Our BAPC projections to 2050 reveal a strikingly divergent future across Asian subregions. Central Asia stands out as the region of greatest concern: age-standardised death rates are projected to increase approximately 4.6-fold from 2023 levels, reaching a median of 2.46 per 100,000 by 2050 (95% PI, 0.93–6.43), with DALY rates projected to rise more than four-fold (from 9.78 to a median of 43.49 per 100,000 [95% PI, 15.68–129.46]). If realised, this trajectory would place Central Asia—currently among the lowest-burden subregions—at a mortality level approaching that of High-income Asia Pacific within three decades, with profound implications for healthcare systems still transitioning from post-Soviet restructuring. In contrast, Southeast Asia is projected to experience only modest increases (death rate +15%, DALY rate +13% from 2023 to 2050), although its absolute burden remains mid-range, and continued surveillance in rapidly ageing Southeast Asian nations such as Thailand, Malaysia, and Vietnam remains warranted [33].

The remaining subregions present a more favourable picture. South Asia is the only subregion projected to achieve sustained declines in both death (−30%) and DALY (−33%) rates by 2050, and East Asia is projected to remain essentially stable (death rate +22%; DALY rate +4%), consistent with its minimal trend change over the past three decades and reflecting sustained economic development, improved healthcare infrastructure, and effective antibiotic stewardship [10]. High-income Asia Pacific, despite beginning from by far the highest baseline, is projected to increase only modestly (death rate +32%; DALY rate +14%), suggesting that even advanced healthcare systems face residual upward pressure, plausibly from extreme population ageing that cannot be fully offset by clinical care.

These findings carry significant implications for GAS vaccine development and implementation strategy. The World Health Organization has identified GAS as a priority pathogen, and multiple vaccine candidates are currently in preclinical and clinical development, including M protein-based peptides (J8, J14), multicomponent subunit vaccines (Combo5, VAX-A1), and conserved non-M protein antigens targeting SpyCEP, SLO, and ScpA [12,13,14]. Our projections suggest that the largest relative mortality reduction from vaccination would be achievable in Central Asia, where the projected trajectory is most ominous; in absolute terms; however, High-income Asia Pacific and Southeast Asia would remain major contributors to regional GAS mortality, and vaccine value assessments should weigh both dimensions. Targeting adults aged 60 years and older—a demographic often excluded from vaccine trials—could substantially reduce iGAS mortality, given the strong age-related risk gradient observed in our analysis [34].

From a health systems perspective, our findings support the integration of GAS prevention into broader non-communicable disease and infectious disease control frameworks for older adults. Enhanced surveillance for iGAS, particularly in long-term care facilities where outbreaks can be devastating, should be prioritised in high-burden countries [35]. Antimicrobial stewardship programmes must be strengthened to combat rising macrolide and clindamycin resistance, which has been documented in Asian GAS isolates [11].

Study limitations warrant consideration. First, GBD estimates rely on modelling where primary data are sparse, particularly in low-income settings with limited vital registration and microbiological surveillance. Second, the BAPC model assumes that historical age, period, and cohort effects will persist into the future, an assumption that may be violated by unforeseen public health interventions, pandemics, or healthcare system disruptions. Third, our analysis did not differentiate between specific GAS clinical syndromes (pharyngitis, skin infection, invasive disease, immune-mediated sequelae), which may exhibit distinct epidemiological patterns. Fourth, subregional aggregates are population-weighted means in which the largest countries (e.g., China within East Asia, India within South Asia) dominate; consequently, subregional trends may not represent smaller countries within the same subregion, and the substantial within-subregional heterogeneity should be considered when interpreting subregional projections. Fifth, we did not conduct cause-specific decomposition for acute rheumatic fever and rheumatic heart disease in this study, as standard GBD 2023 public datasets do not provide disaggregated syndrome-level estimates for GAS-attributable outcomes. Consequently, the fractional burden contributed by ARF and RHD could not be directly quantified in the present analysis. We acknowledge this limitation transparently and recommend that future studies prioritise detailed subphenotype decomposition to better characterise GAS-attributable long-term sequelae across Asian populations. Fifth, because the composite indicator includes latency-bearing sequelae–deaths from rheumatic heart disease reflect GAS infection often acquired in childhood or adolescence–the cohort effects estimated by the BAPC model partly characterise the epidemiology of GAS pharyngitis and rheumatic sequelae in earlier birth generations rather than the contemporaneous dynamics of invasive infection in older adults; accordingly, cohort-based projections should not be interpreted as forecasts of invasive-disease incidence alone. Sixth, retrospective validation (fit 1990–2013, predict 2014–2023) achieved 84% empirical coverage of the 95% prediction intervals overall but only 20% for Central Asia, indicating that the intervals are likely underestimated precisely in the subregion with the fastest change; moreover, a 10-year retrospective evaluation cannot validate a 27-year horizon. Projections for Central Asia should therefore be interpreted as scenario continuations of recent trends with substantial, and possibly under-quantified, uncertainty. Finally, the ecological nature of GBD data precludes individual-level risk factor adjustment.

Future research on Group A Streptococcus (GAS) should focus on several key areas. First, disaggregating the GAS burden by clinical syndromes—such as pharyngitis, skin infections, invasive disease, and immune-mediated sequelae—will allow for more accurate estimations of the preventable fraction through vaccination once syndrome-specific estimates are available. Second, the validity of BAPC projections needs reassessment alongside new iterations from the Global Burden of Disease (GBD) study; integrating scenario-based projections that account for factors like accelerated vaccine introduction could enhance the policy relevance of future studies. Third, country-level research that incorporates primary iGAS surveillance data, especially from Central and Southeast Asian nations with incomplete vital registration, is essential for validating and refining existing model-based estimates. From a practical standpoint, the projections provide a quantitative framework for vaccine value assessments, emphasizing the need to prioritize vaccination for adults aged 60 and older in Central and Southeast Asia, which could significantly reduce mortality. Furthermore, these subregional insights will aid in shaping target product profiles and economic models for candidate vaccines such as M protein-based peptides (J8, J14) and multicomponent subunit vaccines (Combo5, VAX-A1) in development [12,13,14].

## 5. Conclusions

This study demonstrates that Asia’s ageing populations face markedly divergent GAS-attributable health burdens. Central Asia is projected to face the largest relative increase in mortality and DALYs by 2050, accompanied by substantial, possibly under-quantified uncertainty, while High-income Asia Pacific carries the highest absolute burden throughout the projection period. These findings provide a critical evidence base for prioritising GAS vaccine development, refining target-population selection—particularly for Central Asia and other high-growth settings—and tailoring regional public health strategies to protect vulnerable older adults across Asia.

## Figures and Tables

**Figure 1:**
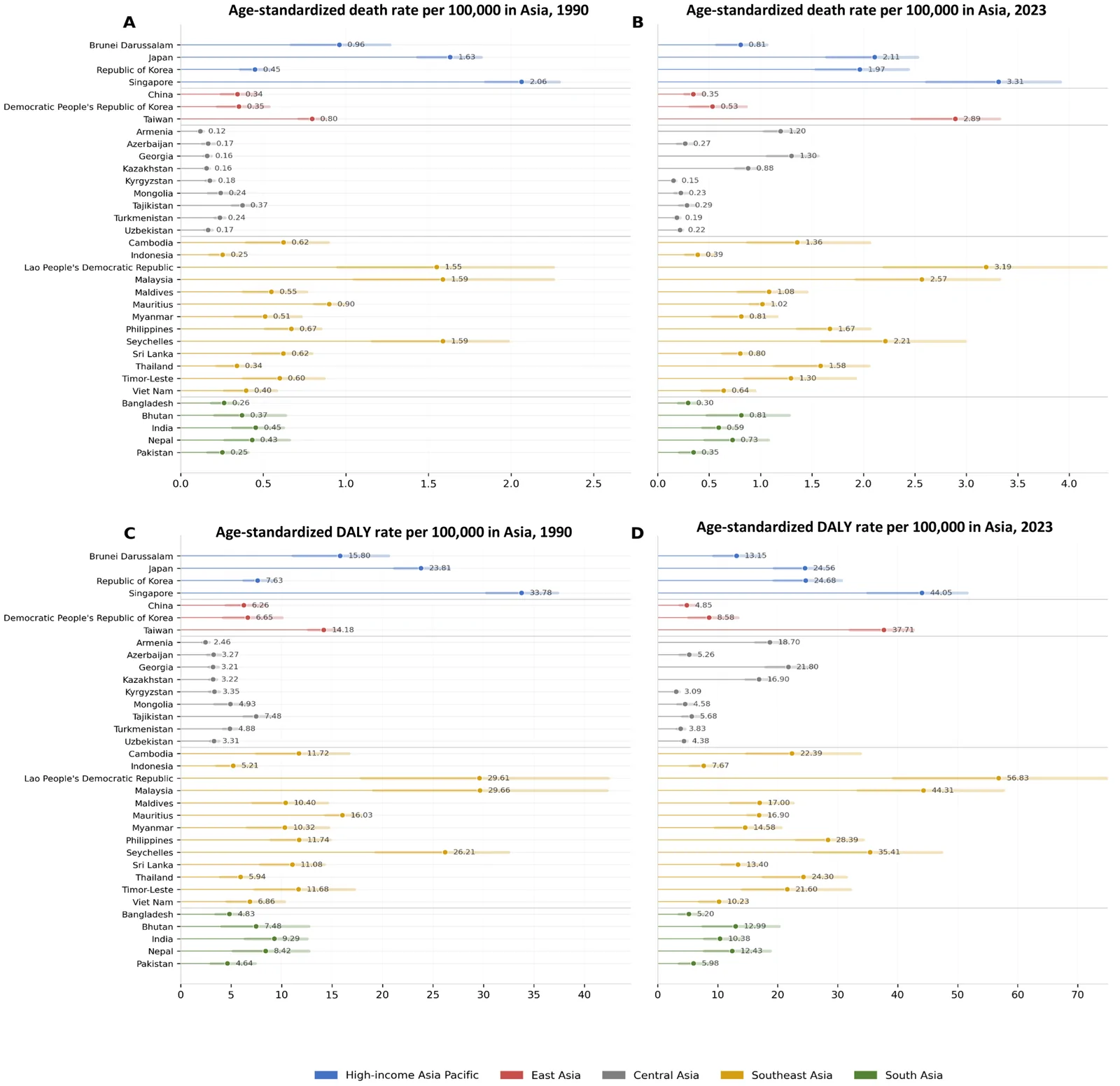
Age-standardised death rates (**A**,**B**) and DALYs (**C**,**D**) for Group A Streptococcus among population aged 60+ years in Asia, in 1990 and 2023. DALY, disability-adjusted life year. Error bars show 95% uncertainty intervals.

**Figure 2:**
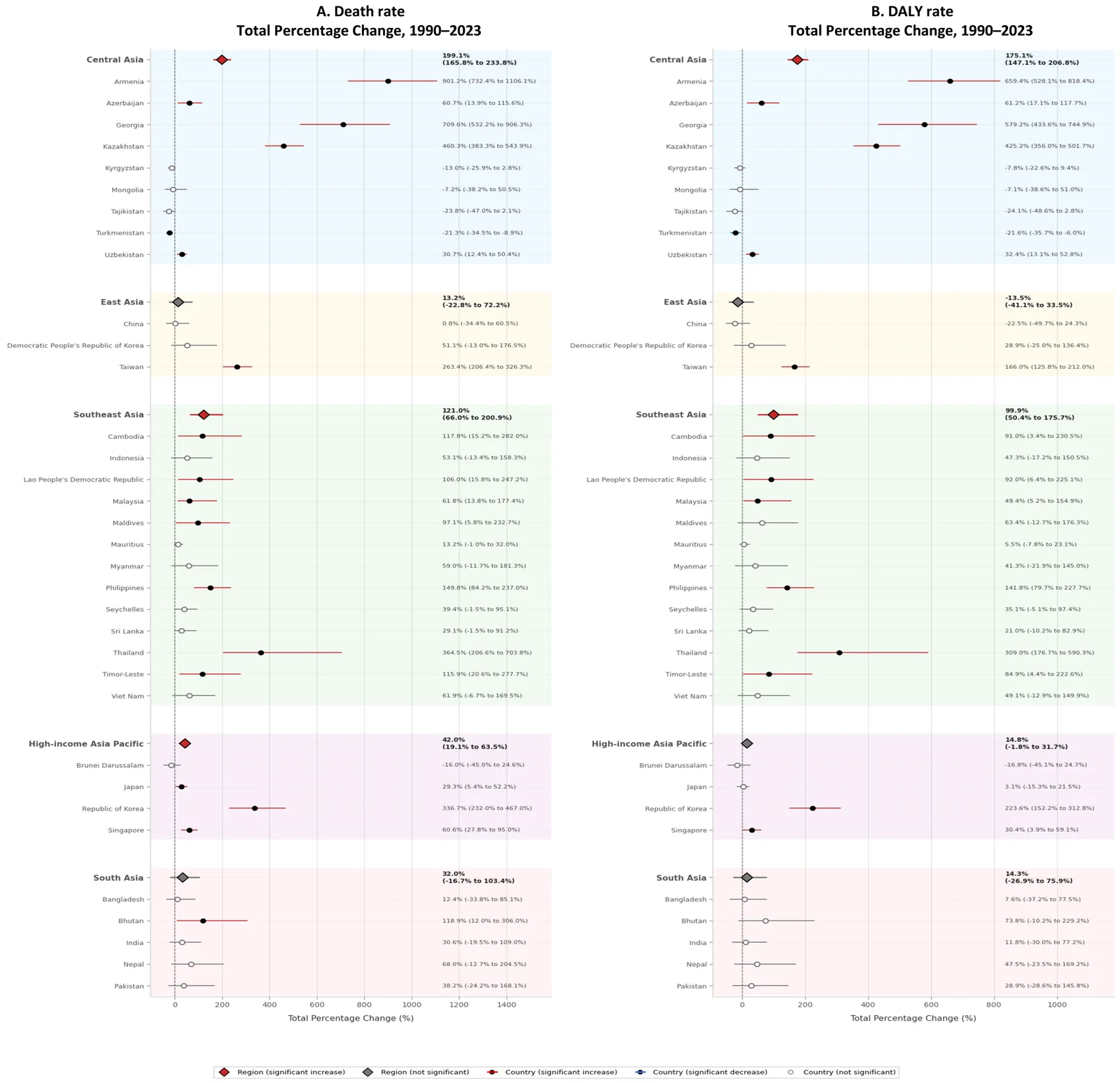
Forest plot of the total percentage change in death rates (**A**) and DALY rates (**B**) among adults ≥60 years, 1990–2023; points represent country-specific estimates with 95% uncertainty intervals, and colours/shapes indicate direction and statistical significance.

**Figure 3:**
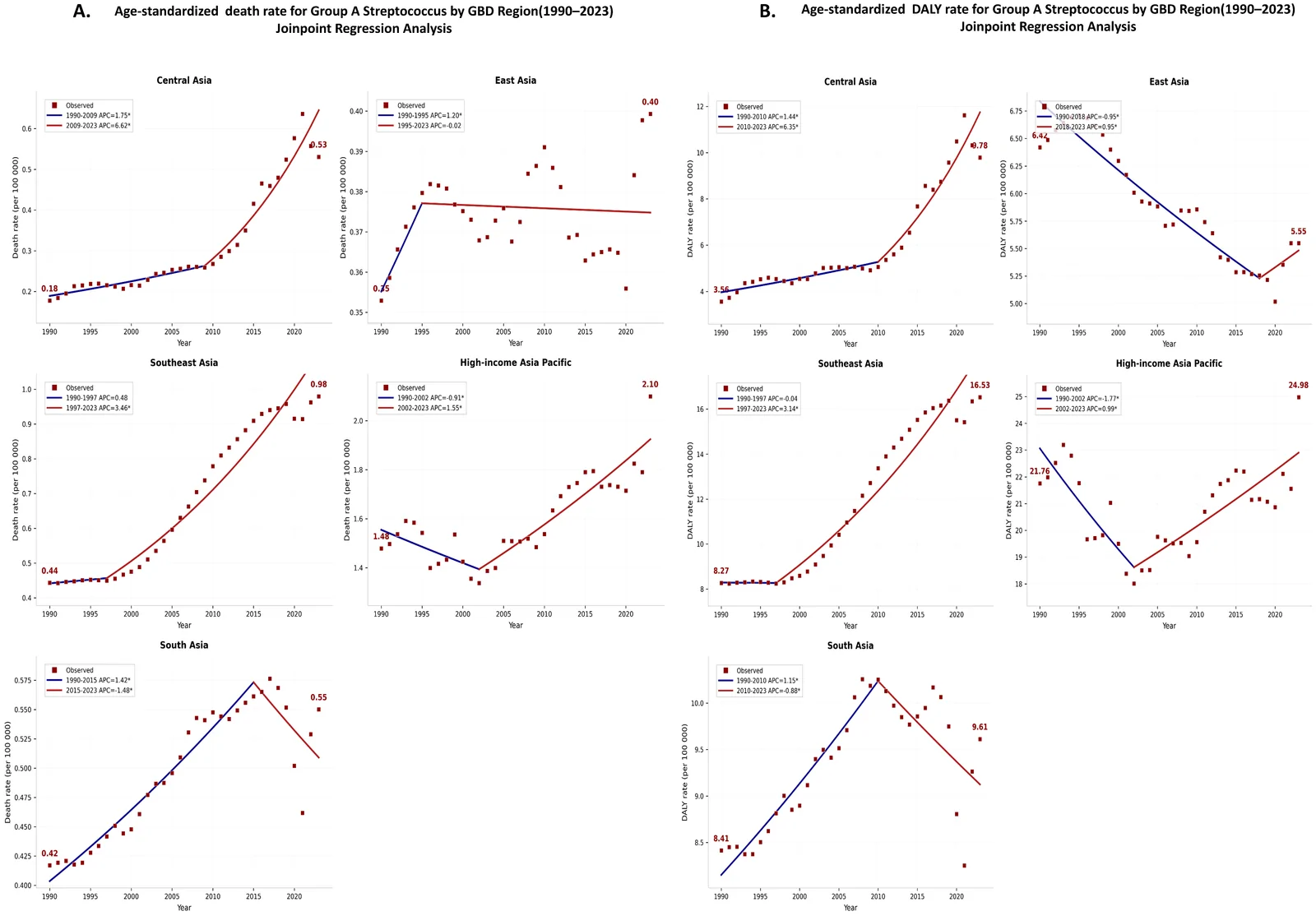
Joinpoint regression analysis of age-standardised death rates (**A**) and age-standardised DALY rates (**B**), 1990–2023, stratified by GBD subregion. * indicates a P value less than 0.05.

**Figure 4:**
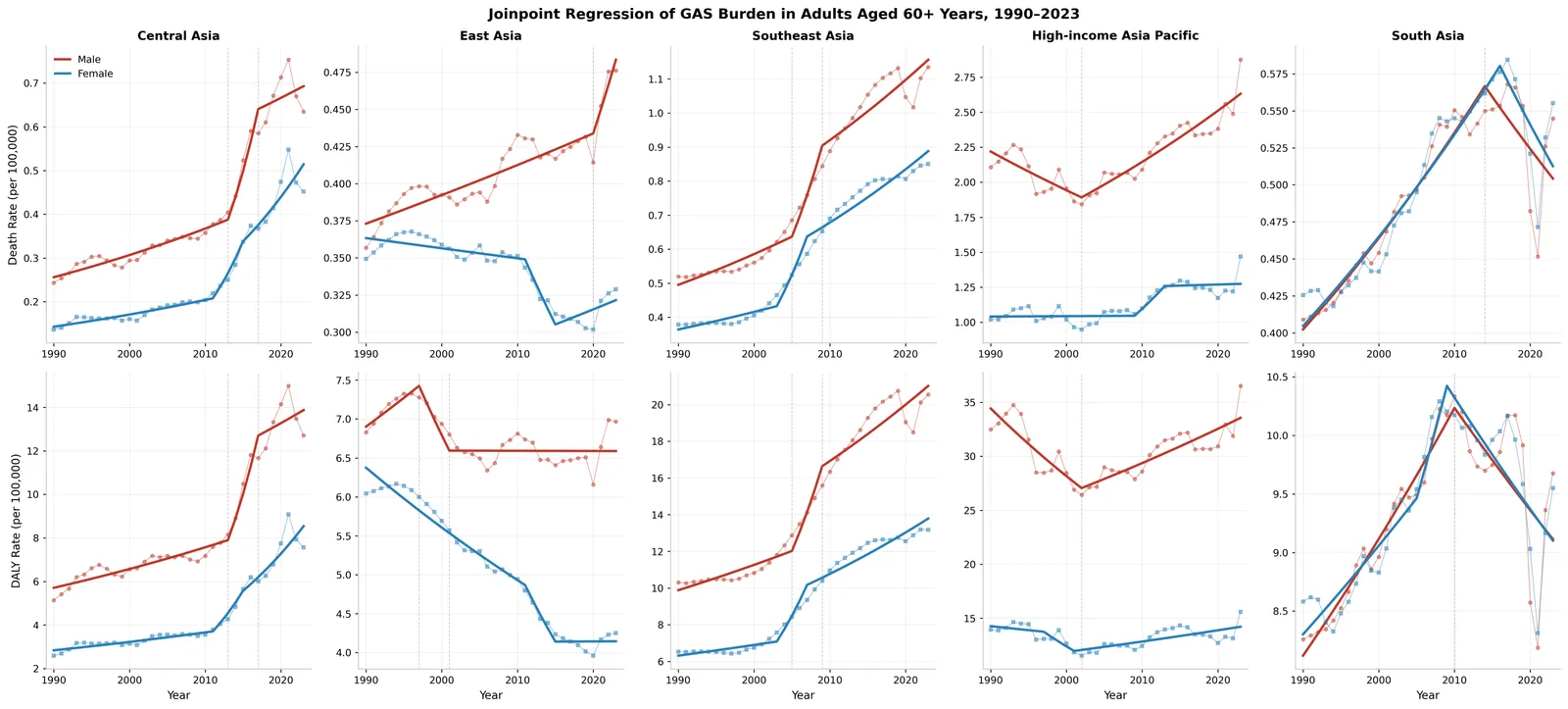
Sex-specific age-standardized death rates (**above**) and DALY rates (**below**) (1990–2023).

**Figure 5:**
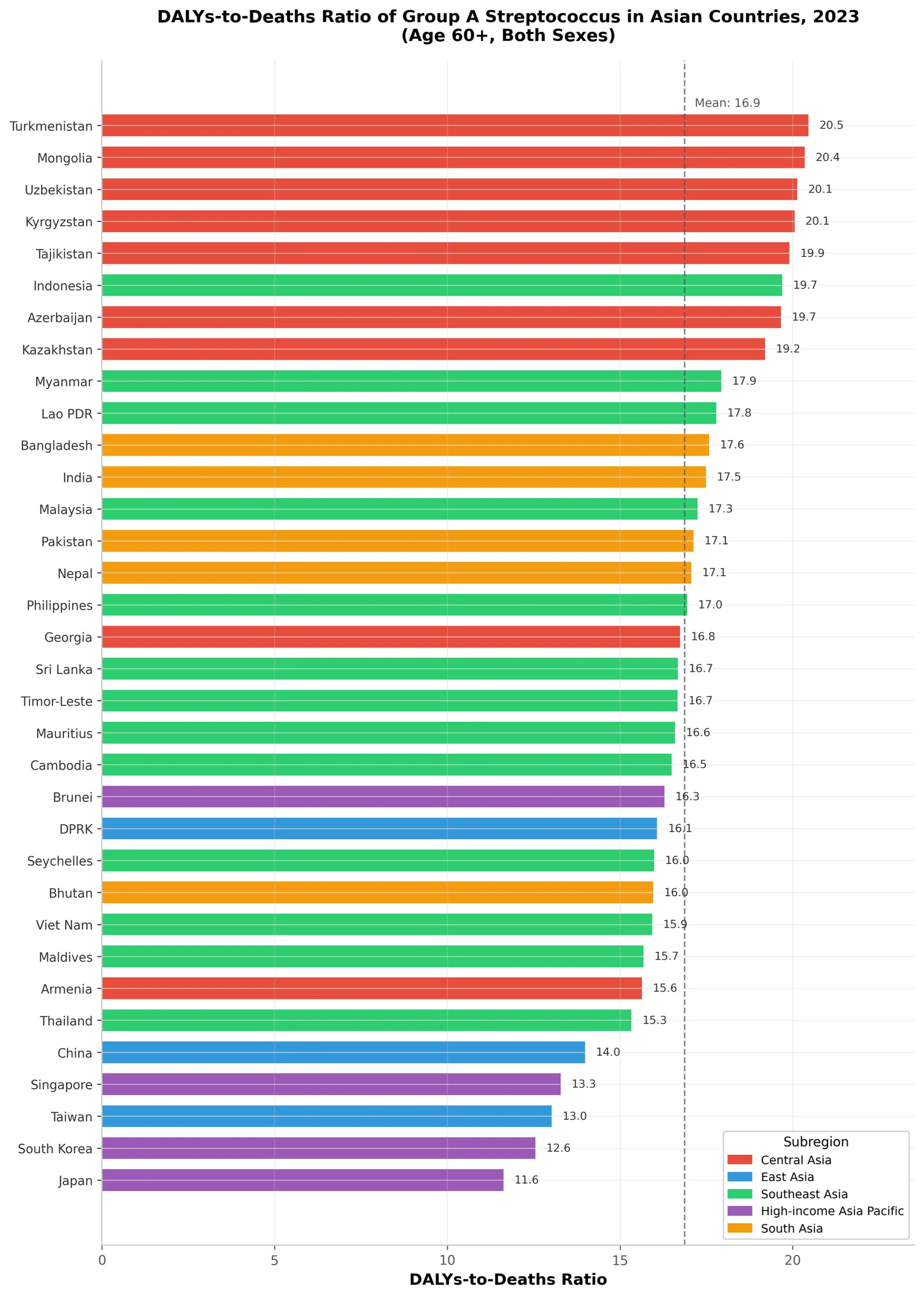
DALYs-to-Deaths Ratio of Group A Streptococcus in Asian countries and territories (Aged 60+ years, Both Sexes).

**Figure 6:**
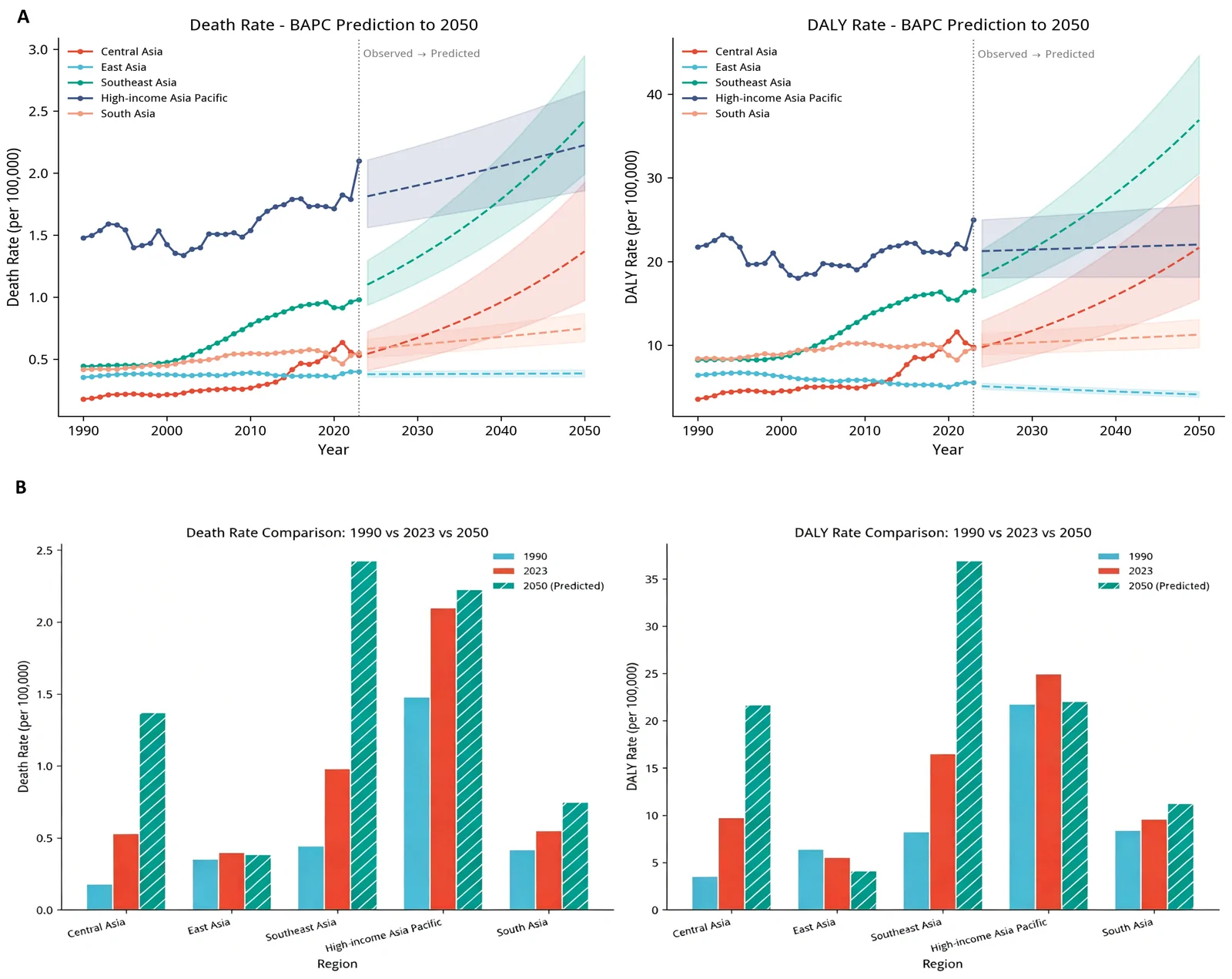
Projected Group A Streptococcus-Attributable Death and DALY Rates in Asian Regions, 2023–2050: A Bayesian Age-Period-Cohort (BAPC) Model Analysis. (**A**). Death rate and DALY Rate BAPC Prediction to 2050. (**B**). Death rate and DALY Rate Comparison.

**Table 1:** Age-standardized GAS-attributable death and DALY rates (per 100,000 population) among adults aged ≥60 years in Asia, 1990 and 2023.

Country/Region	Death Rate 1990 (95% UI)	Death Rate 2023 (95% UI)	Death Rate Change 1990–2023, %	DALY Rate 1990 (95% UI)	DALY Rate 2023 (95% UI)	DALY Rate Change 1990–2023, %	DALY-to-Death Ratio, 2023	M: F Death Ratio, 2023	Male Death Rate 2023	Female Death Rate 2023
East Asia	0.35 (0.26–0.47)	0.40 (0.31–0.51)	+13%	6.42 (4.68–8.47)	5.55 (4.36–7.04)	−14%	13.90	1.45	0.48	0.33
Southeast Asia	0.44 (0.32–0.56)	0.98 (0.84–1.17)	+121%	8.27 (6.17–10.54)	16.53 (14.11–19.36)	+100%	16.87	1.33	1.14	0.85
Central Asia	0.18 (0.16–0.20)	0.53 (0.47–0.58)	+199%	3.56 (3.22–3.91)	9.78 (8.75–10.63)	+175%	18.44	1.40	0.63	0.45
South Asia	0.42 (0.29–0.56)	0.55 (0.41–0.74)	+32%	8.41 (5.93–11.33)	9.61 (7.25–12.63)	+14%	17.47	0.98	0.54	0.56
High-income Asia Pacific	1.48 (1.30–1.65)	2.10 (1.72–2.46)	+42%	21.76 (19.44–24.32)	24.98 (20.82–28.98)	+15%	11.90	1.96	2.87	1.47
Singapore	2.06 (1.85–2.29)	3.31 (2.61–3.91)	+61%	33.78 (30.31–37.33)	44.05 (34.99–51.54)	+30%	13.29	1.20	3.62	3.01
Lao People’s Democratic Republic	1.55 (0.95–2.26)	3.19 (2.20–4.49)	+106%	29.61 (17.91–42.38)	56.83 (39.31–79.36)	+92%	17.80	1.05	3.27	3.12
Taiwan *	0.80 (0.71–0.88)	2.89 (2.47–3.32)	+263%	14.18 (12.65–15.81)	37.71 (32.07–42.59)	+166%	13.03	1.83	3.83	2.09
Malaysia	1.59 (1.05–2.26)	2.57 (1.93–3.32)	+62%	29.66 (19.12–42.25)	44.31 (33.35–57.66)	+49%	17.25	1.16	2.76	2.38
Seychelles	1.59 (1.16–1.98)	2.21 (1.59–2.99)	+39%	26.21 (19.33–32.50)	35.41 (26.00–47.32)	+35%	16.00	1.26	2.47	1.97
Japan	1.63 (1.43–1.82)	2.11 (1.64–2.53)	+29%	23.81 (21.19–26.60)	24.56 (19.40–28.98)	+3%	11.64	2.16	3.01	1.39
Republic of Korea	0.45 (0.36–0.55)	1.97 (1.54–2.44)	+337%	7.63 (6.25–9.25)	24.68 (19.35–30.60)	+224%	12.56	1.50	2.40	1.60
Philippines	0.67 (0.51–0.85)	1.67 (1.36–2.07)	+150%	11.74 (8.90–14.78)	28.39 (23.07–34.26)	+142%	16.96	1.20	1.84	1.54
Thailand	0.34 (0.22–0.47)	1.58 (1.13–2.05)	+364%	5.94 (3.94–8.21)	24.30 (17.59–31.44)	+309%	15.34	1.41	1.88	1.34
Cambodia	0.62 (0.40–0.89)	1.36 (0.87–2.06)	+118%	11.72 (7.49–16.67)	22.39 (14.80–33.79)	+91%	16.50	0.85	1.23	1.45
Georgia	0.16 (0.14–0.18)	1.30 (1.06–1.56)	+710%	3.21 (2.79–3.73)	21.80 (17.97–26.00)	+579%	16.75	1.39	1.57	1.13
Timor-Leste	0.60 (0.38–0.87)	1.30 (0.85–1.92)	+116%	11.68 (7.34–17.20)	21.60 (14.07–32.11)	+85%	16.68	1.08	1.34	1.25
Armenia	0.12 (0.10–0.14)	1.20 (1.04–1.38)	+901%	2.46 (2.14–2.82)	18.70 (16.29–21.56)	+659%	15.65	1.22	1.34	1.09
Maldives	0.55 (0.38–0.76)	1.08 (0.78–1.45)	+97%	10.40 (7.11–14.55)	17.00 (12.15–22.57)	+63%	15.70	1.10	1.13	1.03
Mauritius	0.90 (0.81–1.00)	1.02 (0.90–1.14)	+13%	16.03 (14.36–17.84)	16.90 (14.99–18.85)	+5%	16.60	1.45	1.23	0.85
Kazakhstan	0.16 (0.14–0.17)	0.88 (0.75–1.00)	+460%	3.22 (2.88–3.55)	16.90 (14.69–19.02)	+425%	19.21	1.67	1.16	0.69
Brunei Darussalam	0.96 (0.67–1.27)	0.81 (0.57–1.06)	−16%	15.80 (11.11–20.61)	13.15 (9.30–17.30)	−17%	16.30	1.62	1.01	0.62
Myanmar	0.51 (0.33–0.73)	0.81 (0.53–1.16)	+59%	10.32 (6.60–14.65)	14.58 (9.57–20.58)	+41%	17.94	1.46	0.99	0.68
Bhutan	0.37 (0.21–0.63)	0.81 (0.48–1.28)	+119%	7.48 (4.05–12.71)	12.99 (7.50–20.21)	+74%	15.97	0.76	0.71	0.92
Sri Lanka	0.62 (0.43–0.79)	0.80 (0.63–1.03)	+29%	11.08 (7.92–14.20)	13.40 (10.59–16.86)	+21%	16.69	1.54	1.00	0.65
Nepal	0.43 (0.27–0.66)	0.73 (0.46–1.08)	+68%	8.42 (5.20–12.69)	12.43 (7.73–18.77)	+48%	17.07	0.91	0.69	0.76
Viet Nam	0.40 (0.26–0.58)	0.64 (0.43–0.94)	+62%	6.86 (4.61–10.28)	10.23 (6.95–14.88)	+49%	15.95	1.40	0.77	0.55
India	0.45 (0.31–0.62)	0.59 (0.44–0.80)	+31%	9.29 (6.37–12.53)	10.38 (7.75–13.92)	+12%	17.50	0.94	0.58	0.61
Democratic People’s Republic of Korea	0.35 (0.22–0.53)	0.53 (0.31–0.86)	+51%	6.65 (4.22–10.02)	8.58 (5.16–13.36)	+29%	16.09	0.83	0.48	0.57
Indonesia	0.25 (0.17–0.36)	0.39 (0.27–0.55)	+53%	5.21 (3.56–7.45)	7.67 (5.34–10.96)	+47%	19.71	1.67	0.49	0.29
China	0.34 (0.25–0.47)	0.35 (0.26–0.46)	+1%	6.26 (4.47–8.37)	4.85 (3.68–6.37)	−22%	14.00	1.43	0.41	0.29
Pakistan	0.25 (0.16–0.41)	0.35 (0.21–0.53)	+38%	4.64 (3.00–7.39)	5.98 (3.58–9.24)	+29%	17.14	3.33	0.51	0.15
Bangladesh	0.26 (0.19–0.35)	0.30 (0.20–0.42)	+12%	4.83 (3.47–6.41)	5.20 (3.55–7.69)	+8%	17.59	0.95	0.29	0.30
Tajikistan	0.37 (0.31–0.46)	0.29 (0.21–0.37)	−24%	7.48 (6.25–9.08)	5.68 (4.12–7.57)	−24%	19.92	1.54	0.35	0.23
Azerbaijan	0.17 (0.13–0.20)	0.27 (0.19–0.35)	+61%	3.27 (2.64–3.95)	5.26 (3.70–6.88)	+61%	19.67	1.56	0.33	0.21
Mongolia	0.24 (0.17–0.34)	0.23 (0.16–0.29)	−7%	4.93 (3.36–6.88)	4.58 (3.32–5.85)	−7%	20.36	1.95	0.32	0.16
Uzbekistan	0.17 (0.15–0.19)	0.22 (0.19–0.24)	+31%	3.31 (2.95–3.78)	4.38 (3.89–4.91)	+32%	20.14	1.51	0.27	0.18
Turkmenistan	0.24 (0.21–0.27)	0.19 (0.16–0.22)	−21%	4.88 (4.24–5.56)	3.83 (3.19–4.51)	−22%	20.47	1.67	0.24	0.15
Kyrgyzstan	0.18 (0.15–0.20)	0.15 (0.13–0.18)	−13%	3.35 (2.87–3.83)	3.09 (2.68–3.57)	−8%	20.07	1.68	0.20	0.12

Data are presented as point estimates (95% uncertainty interval). Bold rows indicate GBD subregional aggregates. DALY, disability-adjusted life year; UI, uncertainty interval. * A province in China.

**Table 2:** Decomposition of GAS-attributable DALYs into years of life lost (YLLs) and years lived with disability (YLDs) among adults aged ≥60 years, 2023.

Location	YLLs, n	YLDs, n	YLL Share, %	YLD Share, %	YLL Rate	YLD Rate	DALY Rate
East Asia	16,901.8	102.66	99.4	0.60	5.52	0.034	5.55
Southeast Asia	14,543.9	29.22	99.8	0.20	16.50	0.033	16.53
Central Asia	1095.0	1.73	99.8	0.16	9.77	0.015	9.78
South Asia	17,997.1	125.64	99.3	0.69	9.55	0.067	9.61
High-income Asia Pacific	14,739.4	13.42	99.9	0.09	24.96	0.023	24.98
Armenia	140.0	0.21	99.9	0.15	18.68	0.027	18.70
Azerbaijan	77.9	0.17	99.8	0.22	5.25	0.012	5.26
Bangladesh	816.9	6.80	99.2	0.83	5.16	0.043	5.20
Bhutan	10.1	0.04	99.6	0.38	12.94	0.049	12.99
Brunei Darussalam	6.7	0.00	99.9	0.07	13.14	0.010	13.15
Cambodia	378.7	0.71	99.8	0.19	22.35	0.042	22.39
China	14,282.4	95.50	99.3	0.66	4.82	0.032	4.85
Democratic People’s Republic of Korea	351.3	1.30	99.6	0.37	8.54	0.032	8.58
Georgia	182.5	0.27	99.9	0.15	21.76	0.032	21.80
India	15,832.9	108.43	99.3	0.68	10.31	0.071	10.38
Indonesia	2401.4	6.20	99.7	0.26	7.65	0.020	7.67
Japan	10,825.3	10.78	99.9	0.10	24.54	0.024	24.56
Kazakhstan	459.6	0.56	99.9	0.12	16.88	0.020	16.90
Kyrgyzstan	20.1	0.08	99.6	0.38	3.08	0.012	3.09
Lao People’s Democratic Republic	326.0	0.22	99.9	0.07	56.79	0.037	56.83
Malaysia	1803.6	2.31	99.9	0.13	44.25	0.057	44.31
Maldives	6.3	0.01	99.9	0.15	16.97	0.025	17.00
Mauritius	42.6	0.08	99.8	0.18	16.87	0.031	16.90
Mongolia	12.9	0.03	99.8	0.23	4.57	0.010	4.58
Myanmar	893.9	1.10	99.9	0.12	14.56	0.018	14.58
Nepal	394.3	1.80	99.5	0.45	12.37	0.057	12.43
Pakistan	942.9	8.57	99.1	0.90	5.93	0.054	5.98
Philippines	2930.7	4.85	99.8	0.17	28.35	0.047	28.39
Republic of Korea	3372.6	2.24	99.9	0.07	24.66	0.016	24.68
Seychelles	5.6	0.01	99.9	0.14	35.36	0.049	35.41
Singapore	534.8	0.39	99.9	0.07	44.01	0.032	44.05
Sri Lanka	531.7	1.47	99.7	0.28	13.36	0.037	13.40
Taiwan	2268.0	5.86	99.7	0.26	37.61	0.097	37.71
Tajikistan	39.6	0.08	99.8	0.21	5.67	0.012	5.68
Thailand	3759.1	8.45	99.8	0.22	24.24	0.055	24.30
Timor-Leste	22.8	0.03	99.9	0.14	21.57	0.030	21.60
Turkmenistan	20.0	0.05	99.7	0.27	3.82	0.010	3.83
Uzbekistan	142.3	0.28	99.8	0.20	4.37	0.009	4.38
Viet Nam	1423.6	3.75	99.7	0.26	10.21	0.027	10.23

Estimates extracted from GBD 2023 (GHDx) with measures of YLLs and YLDs, REI “Group A Streptococcus” (rei_id 630), all causes (cause_id 294), age of 60+ years, both sexes, 2023. YLL and YLD rates sum exactly to the DALY rates reported in Table 1. GAS-attributable YLLs and YLDs in this age group are carried entirely by lower respiratory infections (cause_id 322). DALY, disability-adjusted life year; YLD, years lived with disability; and YLL, years of life lost.

**Table 3:** BAPC projections of age-standardised GAS-attributable death and DALY rates (per 100,000 population) among adults aged ≥60 years in five Asian subregions, 2023–2050.

Subregion	2023 Observed, Mean (95% UI)	2024 Projected, Median (95% PI)	2050 Projected, Median (95% PI)	Change 2023–2050, %
**(A) Death rate**				
East Asia	0.40 (0.31–0.51)	0.39 (0.33–0.46)	0.49 (0.18–1.41)	+22%
Southeast Asia	0.98 (0.84–1.17)	0.98 (0.86–1.12)	1.13 (0.40–3.16)	+15%
Central Asia	0.53 (0.47–0.58)	0.66 (0.62–0.71)	2.46 (0.93–6.43)	+364%
South Asia	0.55 (0.41–0.74)	0.51 (0.42–0.63)	0.39 (0.11–1.30)	−29%
High-income Asia Pacific	2.10 (1.72–2.46)	1.92 (1.72–2.16)	2.77 (1.18–6.37)	+32%
**(B) DALY rate**				
East Asia	5.55 (4.36–7.04)	5.33 (4.61–6.24)	5.76 (2.22–16.33)	+4%
Southeast Asia	16.53 (14.11–19.36)	16.53 (14.58–18.70)	18.63 (6.27–49.93)	+13%
Central Asia	9.78 (8.75–10.63)	12.05 (11.25–12.89)	43.49 (15.68–129.46)	+344%
South Asia	9.61 (7.25–12.63)	8.98 (7.47–10.90)	6.43 (1.97–21.33)	−33%
High-income Asia Pacific	24.98 (20.82–28.98)	22.86 (20.61–25.62)	28.55 (12.39–68.01)	+14%

DALY, disability-adjusted life year; PI, prediction interval; and UI, uncertainty interval. All reported estimates were generated under a single analytical protocol with effective degrees of freedom of 6, using 2000 replicates and a fixed random seed, thereby ensuring internal consistency across every result. Projected rates are reported as the median of the posterior predictive distribution, with uncertainty expressed as 95% PI (2.5th–97.5th percentiles).

## Data Availability

All data used in this study are publicly available from the Global Burden of Disease Study 2023 database (https://ghdx.healthdata.org/gbd-2023, accessed on 1 January 2026).

## References

[1] Ralph A.P., Carapetis J.R. (2013). Group A streptococcal diseases and their global burden. Curr. Top. Microbiol. Immunol..

[2] Carapetis J.R., Steer A.C., Mulholland E.K., Weber M. (2005). The global burden of group A streptococcal diseases. Lancet Infect. Dis..

[3] Miller K.M., Carapetis J.R., Van Beneden C.A., Cadarette D., Daw J.N., Moore H.C., Bloom D.E., Cannon J.W. (2022). The global burden of sore throat and group A Streptococcus pharyngitis: A systematic review and meta-analysis. eClinicalMedicine.

[4] NCBI Bookshelf (2024). Group A Streptococcal Infections—StatPearls. https://www.ncbi.nlm.nih.gov/books/NBK559240/.

[5] Steer A.C., Lamagni T., Curtis N., Carapetis J.R. (2012). Invasive group A streptococcal disease: Epidemiology, pathogenesis and management. Drugs.

[6] Andrews R.M., McCarthy J., Carapetis J.R., Currie B.J. (2009). Skin disorders, including pyoderma, scabies, and tinea infections. Pediatr. Clin. North Am..

[7] Lamagni T.L., Efstratiou A., Vuopio-Varkila J., Jasir A., Schalén C. (2005). Strep-EURO Study Group. The epidemiology of severe Streptococcus pyogenes associated disease in Europe. Euro Surveill..

[8] Castro S.A., Dorfmueller H.C. (2021). A brief review on Group A Streptococcus pathogenesis and vaccine development. R Soc. Open Sci..

[9] Cannon J.W., Zhung J., Bennett J., Moreland N.J., Baker M.G., Geelhoed E., Fraser J., Carapetis J.R., Jack S. (2021). The economic and health burdens of diseases caused by group A Streptococcus in New Zealand. Int. J. Infect. Dis..

[10] Sati H., Carrara E., Savoldi A., Hansen P., Garlasco J., Campagnaro E., Boccia S., Castillo-Polo J.A., Magrini E., Garcia-Vello P. (2025). The WHO bacterial priority pathogens list 2024: A prioritisation study to guide research, development, and public health strategies against antimicrobial resistance. Lancet Infect. Dis..

[11] Craik N., Hla T., Cannon J., Moore H., Carapetis J.R., Sanyahumbi A., Ferretti J.J., Stevens D.L., Fischetti V.A. (2024). Global disease burden of Streptococcus pyogenes. Streptococcus Pyogenes: Basic Biology to Clinical Manifestations.

[12] Strep A Vaccine Global Consortium (2019). Paving the Way for a Strep A Vaccine That Benefits the World.

[13] White A. (2018). WHO Resolution on rheumatic heart disease. Eur. Heart J..

[14] Brusco N.K., Oliver J., McMinn A., Steer A., Crawford N. (2021). The cost of care for children hospitalized with invasive Group A Streptococcal Disease in Australia. BMC Health Serv. Res..

[15] United Nations, Department of Economic and Social Affairs, Population Division (2022). World Population Prospects 2022. https://population.un.org/wpp/.

[16] United Nations, Department of Economic and Social Affairs, Population Division (2023). World Population Ageing 2023. https://www.un.org/development/desa/pd/.

[17] GBD 2019 Diseases and Injuries Collaborators (2020). Global burden of 369 diseases and injuries in 204 countries and territories, 1990–2019: A systematic analysis for the Global Burden of Disease Study 2019. Lancet.

[18] GBD 2023 Disease and Injury and Risk Factor Collaborators (2025). Burden of 375 diseases and injuries, risk-attributable burden of 88 risk factors, and healthy life expectancy in 204 countries and territories, including 660 subnational locations, 1990-2023: A systematic analysis for the Global Burden of Disease Study 2023. Lancet.

[19] Bhutta Z.A. (2025). Global Burden of Disease 2023: Challenges and opportunities for a growing collaboration. PLoS Med..

[20] Schmid V.J., Held L. (2017). Bayesian Age-Period-Cohort Modeling and Prediction—BAMP. J. Stat. Softw..

[21] Riebler A., Held L. (2017). Projecting the future burden of cancer: Bayesian age-period-cohort analysis with integrated nested Laplace approximations. Biom. J..

[22] GBD 2019 Demographics Collaborators (2020). Global age-sex-specific fertility, mortality, healthy life expectancy (HALE), and population estimates in 204 countries and territories, 1950–2019: A comprehensive demographic analysis for the Global Burden of Disease Study 2019. Lancet.

[23] Kim H.J., Fay M.P., Feuer E.J., Midthune D.N. (2000). Permutation tests for joinpoint regression with applications to cancer rates. Stat. Med..

[24] National Cancer Institute *Joinpoint Regression Program, Version 5.2.0*; Statistical Research and Applications Branch, National Cancer Institute, 2023. https://surveillance.cancer.gov/joinpoint/.

[25] Clegg L.X., Hankey B.F., Tiwari R., Feuer E.J., Edwards B.K. (2009). Estimating average annual per cent change in trend analysis. Stat. Med..

[26] Rue H., Martino S., Chopin N. (2009). Approximate Bayesian inference for latent Gaussian models by using integrated nested Laplace approximations. J. R Stat. Soc. Ser. B Stat. Methodol..

[27] Lindgren F., Rue H. (2015). Bayesian spatial modelling with R-INLA. J. Stat. Softw..

[28] Moore H.C., Cannon J.W., Kaslow D.C., Lamagni T., Bowen A.C., Miller K.M., Cherian T., Carapetis J.R., Van Beneden C.A. (2022). A systematic framework for prioritizing burden of disease data required for vaccine development and implementation: The case for Group A Streptococcal diseases. Clin. Infect. Dis..

[29] Ibrahim F., Khan T., Pujalte G.G.A. (2015). Bacterial skin infections. Prim. Care.

[30] Moore H.C., Miller K.M., Carapetis J.R., Van Beneden C.A. (2022). Harmonizing surveillance methodologies for group A streptococcal diseases. Open Forum Infect. Dis..

[31] Zhang L., Tong Z., Han R., Li K., Zhang X., Yuan R. (2023). Spatiotemporal trends in global burden of rheumatic heart disease and associated risk factors from 1990 to 2019. Int. J. Cardiol..

[32] Sims Sanyahumbi A., Colquhoun S., Wyber R., Carapetis J.R., Ferretti J.J., Stevens D.L., Fischetti V.A. (2024). Global disease burden of group A streptococcus. Streptococcus Pyogenes: Basic Biology to Clinical Manifestations.

[33] Belete M.A., Toth I., Toth I., Skwarczynski M. (2026). Rational selection of antigenic targets for Group A Streptococcus vaccine: Updates, challenges and opportunities. npj Vaccines.

[34] Miller K.M., Lamagni T., Cherian T., Cannon J.W., Parks T., Adegbola R.A., Pickering J., Barnett T., Engel M.E., Manning L. (2022). Standardization of Epidemiological Surveillance of Invasive Group A Streptococcal Infections. Open Forum Infect. Dis..

[35] Miller K.M., Carapetis J.R., Cherian T., Hay R., Marks M., Pickering J., Cannon J.W., Lamagni T., Romani L., Moore H.C. (2022). Standardization of Epidemiological Surveillance of Group A Streptococcal Impetigo. Open Forum Infect. Dis..

